# Efficient computation of motif discovery on Intel Many Integrated Core (MIC) Architecture

**DOI:** 10.1186/s12859-018-2276-1

**Published:** 2018-08-13

**Authors:** Shaoliang Peng, Minxia Cheng, Kaiwen Huang, YingBo Cui, Zhiqiang Zhang, Runxin Guo, Xiaoyu Zhang, Shunyun Yang, Xiangke Liao, Yutong Lu, Quan Zou, Benyun Shi

**Affiliations:** 1 College of Computer Science and Electronic Engineering & National Supercomputing Centre in Changsha, Hunan University, Changsha, 410082 China; 2 School of Computer Science, National University of Defense Technology, Changsha, 410073 China; 3 National Supercomputer Center in Guangzhou, Guangzhou, 510275 China; 4 School of Computer Science and Technology, Tianjin University, Tianjin, 300350 China; 5 School of Cyberspace, Hangzhou Dianzi University, Hangzhou, 310018 China

**Keywords:** MIC, MEME, Motif discovery, Offload mode

## Abstract

**Background:**

Novel sequence motifs detection is becoming increasingly essential in computational biology. However, the high computational cost greatly constrains the efficiency of most motif discovery algorithms.

**Results:**

In this paper, we accelerate MEME algorithm targeted on Intel Many Integrated Core (MIC) Architecture and present a parallel implementation of MEME called MIC-MEME base on hybrid CPU/MIC computing framework. Our method focuses on parallelizing the starting point searching method and improving iteration updating strategy of the algorithm. MIC-MEME has achieved significant speedups of 26.6 for ZOOPS model and 30.2 for OOPS model on average for the overall runtime when benchmarked on the experimental platform with two Xeon Phi 3120 coprocessors.

**Conclusions:**

Furthermore, MIC-MEME has been compared with state-of-arts methods and it shows good scalability with respect to dataset size and the number of MICs. Source code: https://github.com/hkwkevin28/MIC-MEME.

## Background

Identifying meaningful patterns (i.e., motifs) from biological sequences is an important problem and a major challenge in bioinformatics research. A motif [1] is a nucleotide or amino-acid sequence pattern that recurs in different DNA or protein sequences and has a biological significance. For example, a 12-base-pair motif is a DNA sequence that is repeated several times within the mouse MT-I and other MT promoters and can confer metal regulation [2]. Specific sequence motifs usually mediate a common function. A sequence motif that appears in the exon of a gene may encode the structural motif of a protein [3]. A motif outside of gene exons such as being DNA binding sites for regulatory protein [4] can affect the shape of nucleic acids. And a RNA motif embedded in a common secondary structure may be able to bind ATP [5].

In recent years, it has emerged a large number of computational algorithms for motif discovery which can be categorized into two groups, including word-based (string-based) methods and probabilistic methods [1]. Word-based methods mostly exhaustive enumerate in their computation and probabilistic methods employ probabilistic sequence models where the model parameters are optimized by maximum-likelihood principle or Bayesian inference. Probabilistic methods have the advantage of few parameters and are more appropriate for finding longer or more general motifs especially for prokaryotes, whose motifs are generally longer than eukaryotes.

MEME (Multiple EM for Motif Elicitation) [2] is one of the currently widely-used algorithms based on maximum-likelihood principle for de novo motif discovery [3]. The time complexity of MEME is *O*(*N*^2^ × *L*^2^), where *N* is the number of input sequences and *L* is the average length of each sequence. However, the high computational cost constrains MEME for handling large datasets [4]. To accelerate motif discovery algorithm, most of previous approaches focus on using parallelization on distributed workstations, Graphics Processing Unit (GPU) and Field Programmable Gate Arrays (FPGA). Farouk et al. parallelized the Brute Force algorithm targeted on FPGAs [5]. Marchand et al. scaled Dragon Motif Finder (DMF) to IBM Blue Gene/P using mixed-mode MPI-OpenMP programming [6]. mCUDA–MEME is a parallel implementation of MEME running on multiple GPUs using CUDA programming model [7].

Intel Many Integrated Core (MIC) Architecture [8] is the latest parallel technique which targets high performance computing (HPC) and other parallel computing segments. It is a brand-new many-core architecture that delivers massive thread parallelism, data parallelism, and memory bandwidth in a CPU form factor for high throughput workloads [9]. To accelerate MEME algorithm, we parallelize it targeted on MIC Architecture and presented a parallel implementation of MEME called MIC-MEME based on hybrid CPU/MIC computing framework. We can improve the efficiency of MEME algorithm without losing accuracy. Comparing with the other methods, our method which harnesses the powerful compute capability of MIC is faster and robustness. In this paper, we first introduce sequential MEME algorithm and hybrid CPU/MIC computing framework. Then we present the method to accelerate MEME algorithm which focuses on parallelizing the starting point searching method and improving iteration updating strategy of the algorithm. At last, we discuss the experiments and the performance is presented to evaluate our method.

## Methods

### MEME algorithm

The MEME algorithm [2] is a popular and well established motif discovery algorithm, which extends the expectation maximization (EM) [10] algorithm. Given a set of biopolymer sequences where little or nothing is known in advance about any motifs that may be present, MEME attempts to discover new motifs using a statistical motif model *θ* [11]. A motif model *θ* is a matrix of letter frequencies representing frequency estimates of letters occurring in different positions of a shared motif. Given a motif of width *W* from an alphabet *Σ =* {*X*_1_*,X*_2_*, ...,X*_*N*_}which has *N* letters (eg. the alphabet of DNA is {A,T,C,G}), the size of the matrix *θ* is *N*×(*W+ 1*) and the matrix value *θ*_*i*, *j*_ (*1*≤*i*≤*N* and *0*≤*j*≤*W*) is defined as follows:

1 $$ {\theta}_{i,j}=\Big\{{\displaystyle \begin{array}{c} probabilty\ of\ {X}_i\  appearing\  at\  position\ j\  of\ the\ motif, if\ 1\le j\le W\\ {} probabilty\ of\ {X}_i\  not\ appearing\in the\ motif, if\ j=0\end{array}}\operatorname{} $$

With a set of input sequences, the EM algorithm is carried out from an initial model *θ*^(0)^ which represents a starting point. Then it runs until convergence in order to find the final motif model *θ*^(*q*)^ with maximal posterior probability. Besides, it can just run for a fixed number of iterations before convergence. MEME provides support for three different types of search modes: one occurrence per sequence (OOPS), zero or one occurrence per sequence (ZOOPS), and two component mixture (TCM) [2]. The type of model chosen by a user depends upon prior knowledge concerning the dataset. The OOPS model assumes that there is only one occurrence per sequence of the motif in the dataset, the ZOOPS model is a generalization of OOPS and assumes zero or one occurrence per sequence of the motif, and the TCM model assumes zero or more non-overlapping occurrences of the motif per sequence. Since the OOPS and ZOOPS models are sufficient for most motif finding applications, this paper concentrates on the support for the OOPS and the ZOOPS search models.

During each motif search, MEME does a multi-start search of a given motif width *W* and the search consists of two stages: starting point searching and EM [11].

In the starting point searching stage, MEME iterates over all possible initial models and chooses the initial models with the highest statistical significance. More specifically, MEME converts each *W* length substring occurring in a sequence dataset into a motif model and calculates the weighted log likelihood ratio on different numbers of predicted sites. The potential motif models with the highest weighted log likelihood ratio are selected as starting points for the successive EM stages. Also in this stage, a *P*-value is calculated, which represents the probability of a random string, generated from the background letter frequencies.

In the EM stage, An EM algorithm is performed for a fixed number of iterations or until convergence from each of the highest-scoring initial models and then the best motif model is chosen.

During the starting point searching, Given the input dataset *S =* {*S*_1_*, S*_2_*, ..., S*_*n*_} of *n* biological sequences from *Σ*, and motif width *W*, The following notations are used for the convenience of discussion: *L*_*i*_ denotes the length of sequence *S*_*i*_, ${\overline{s}}_i$ denotes the reverse complement of *S*_*i*_, *S*_*i*, *j*_ denotes the substring starting at position *j* in sequence *S*_*i*_, *S*_*i*_(*j*) denotes the *j*th letter in *S*_*i*_, for *1*≤*i*≤*n* and *0*≤*j*≤*L*_*i*_ − *W*. The starting point searching process primarily consists of four steps for the OOPS and ZOOPS models:

• Calculate the probability score *P*(*S*_*i*, *j*_*, S*_*k*, *l*_) from the forward strand (or *P*(*S*_*i*, *j*_*,* ${\overline{s}}_{k,l}$) from the reverse complement), which represents the probability that a site starts at position *l* in *S*_*k*_ when a site starts at position *j* in *S*_*i*_. The time complexity is *O*(*l*_*i*_*·l*_*k*_) for each sequence pair *S*_*i*_ and *S*_*k*_.

• select the highest-scoring substring *S*_*k*, *maxk*_ (as well as its strand orientation) for each sequence *S*_*k*_. The time complexity is *O*(*l*_*k*_) for each sequence *S*_*k*_.

• Sort the *nsites0* highest-scoring substrings {*S*_*k*, *maxk*_} in decreasing order of scores and determine the potential starting points. The time complexity is *O*(*nlogn*) for OOPS and *O*(*n*^2^*W*) for ZOOPS.

• Update the hash map and starting point heap.

The probability score *P*(*S*_*i*, *j*_, *S*_*k*, *l*_) is computed as:

2 $$ P\left({S}_{i,j},{S}_{k,l}\right)=\sum \limits_{p=0}^{W-1} mat\left[{S}_i\left(j+p\right)\right]\left[{S}_k\left(l+p\right)\right] $$

where *mat* denotes the letter frequency matrix of size *|Σ| × |Σ|*. To reduce computation redundancy, Eq. (2) can be further simplified to Eq. (3), where the computation of the probability scores {*P*(*S*_*i*, *j*_, *S*_*k*, *l*_)} in the *j*th iteration depends on the resulting scores {*P*(*S*_*i*, *j* − 1_, *S*_*k*, *l* − 1_)} in the (*j-1*)th iteration. However, {*P*(*S*_*i*, *j*_, *S*_*k*, 0_)} needs to be computed individually using Eq. (2).

3 $$ {\displaystyle \begin{array}{c}P\left({S}_{i,j},{S}_{k,l}\right)=P\left({S}_{i,j-1},{S}_{k,l-1}\right)+ mat\left[{S}_i\left(j+W-1\right)\right]\left[{S}_k\left(l+W-1\right)\right]\\ {}- mat\left[{S}_i\left(j-1\right)\right]\left[{S}_k\left(l-1\right)\right]\end{array}} $$

**Fig. 1:**
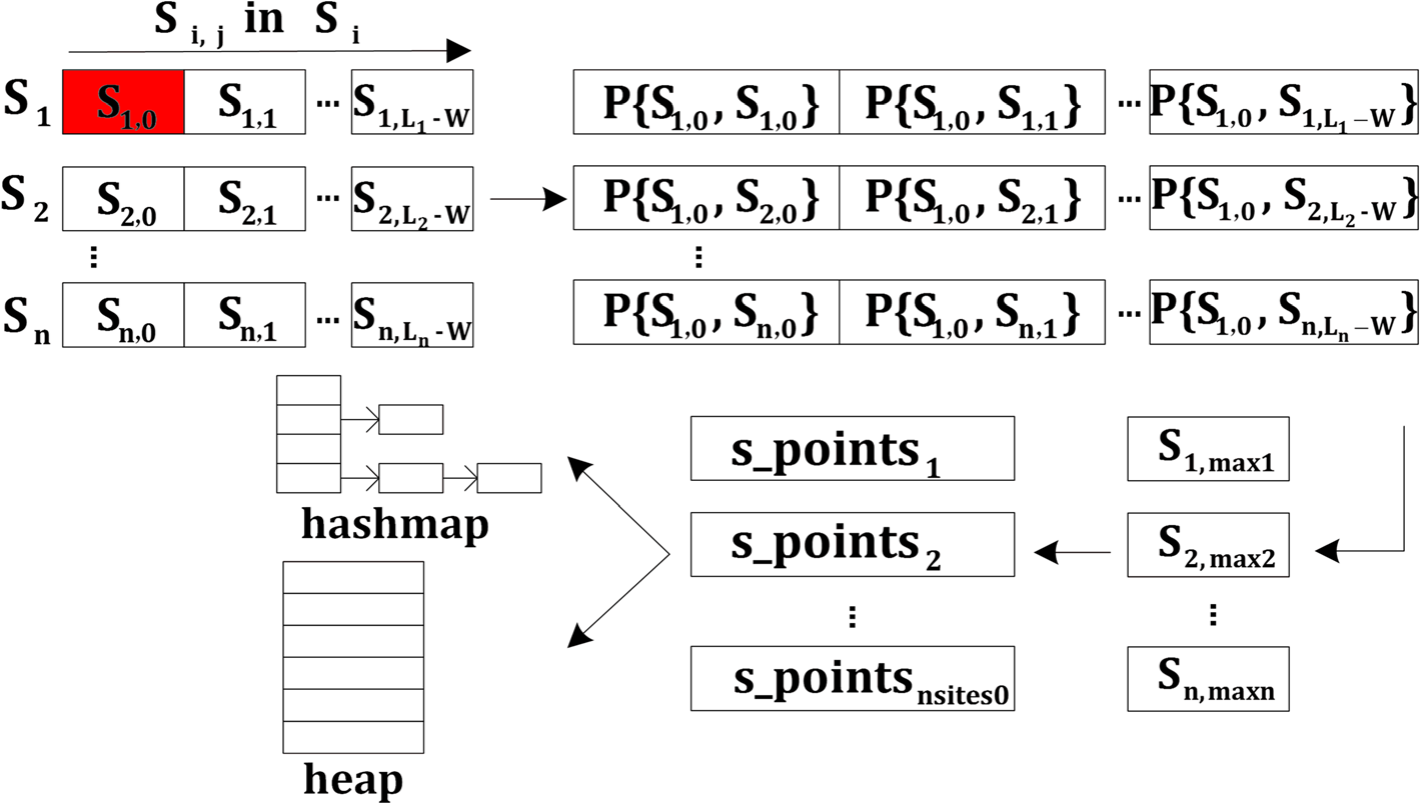
Process of starting point searching algorithm

The detailed algorithm for the starting point searching is illustrated in Algorithm 1. And Fig. 1 shows the process of starting point searching algorithm for the OOPS and ZOOPS models. MEME Suite [12] is an open source implementation of MEME algorithm. The method presented in this paper is based on MEME Suite (version 4.11.2).

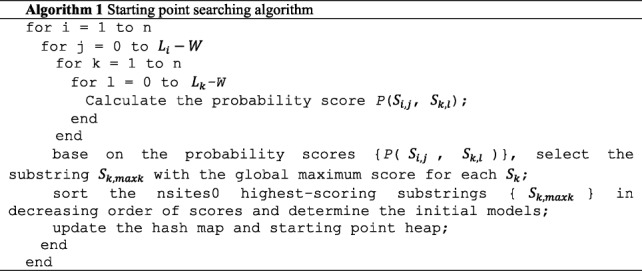



### Hybrid CPU/MIC computing framework

Intel Many Integrated Core (MIC) Architecture [8] is a co-processor computer architecture developed by Intel, which combines many Intel processor cores onto a single chip. The Intel Xeon Phi processor is a bootable host processor that delivers massive parallelism and vectorization to support the most demanding high-performance computing applications. The latest Intel Xeon Phi processor which targets HPC segments provides up to 72 out-of-order cores, Intel Advanced Vector Extensions 512 instructions, and up to 16GB of on-package high-bandwidth memory along with the capacity for 384GB DDR4 platform memory [9].

**Fig. 2:**
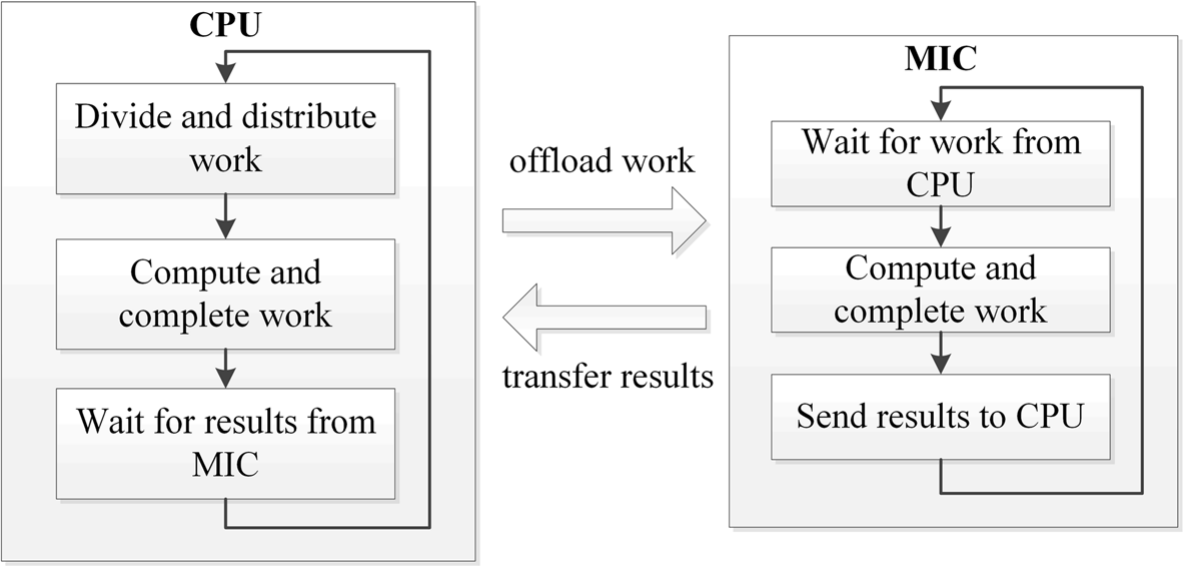
Structure of hybrid CPU/MIC computing framework

MIC provides a processor-centric “offload” model where the program is viewed as running on processors and offloading the work selected to coprocessors. To make full use of computing resources, we have investigated a CPU/MIC collaborated parallel framework which overlaps the computation of the CPU and MIC using offload mode. In this framework, the work is divided into two parts, one runs on CPU and another runs on MIC. The processes on CPU offload the work of MIC to the coprocessor. While the computation of MIC, CPU computes at the same time. After MIC completed its work, the result is transferred to CPU using offload mode. Structure of hybrid CPU/MIC computing framework is present in Fig. 2. In this framework, OpenMP [13] is used for parallelization.

### Improved starting point search strategy

To accelerate MEME algorithm, we analysis sequential MEME with Intel VTune Amplifier. Profiling of MEME algorithm reveals that over 98% of the overall running time is usually spent on the starting point searching stage and the computation of the probability scores {*P*(*S*_*i*, *j*_, *S*_*k*, *l*_)} is the hotspot of the stage. Since the starting point searching stage is the runtime bottleneck of the algorithm, our MIC parallelization approach focuses on parallelizing the starting point searching stage [14]. And in EM stage, the M step and E step of EM algorithm are simply parallelized using OpenMP.

Our parallelization approach of MEME algorithm separates the starting point searching into two parts: probability score computation and highest score sortation.

In probability score computation part, the probability scores {*P*(*S*_*i*, *j*_, *S*_*k*, *l*_)} are calculated in parallel. We take advantage of the fact that for a given *W* length substring *S*_*i*, *j*_ (*1*≤*i*≤*n* and *0*≤*j*≤*L*_*i*_*-W*), the computation of scores {*P*(*S*_*i*, *j*_, *S*_*k*, *l*_)} are independent of each other for any sequence *S*_*k*_ (*1*≤*k*≤*n* and *0*≤*l*≤*L*_*k*_*-W*). Let several threads comprise a thread group. Each thread group is assigned to compute the scores of all *W* length substrings in one sequence *S*_*i*_against any sequence *S*_*k*_. For one substring *S*_*i*, *j*_ in sequence *S*_*i*_, all threads in the thread group compute the scores against all the sequences, where for each sequence *S*_*k*_, the set of all the *W* length substrings {*S*_*k*, *l*_} are roughly equally divided and distributed to *m* threads. After calculating the probability score, each thread *p* (*1*≤*p*≤*m*) selects the local highest-scoring substring ${S}_{k,{maxk}_{localp}}$ for each set of substrings {*S*_*k*, *l*_}, which will be used in highest score sortation part.

**Fig. 3:**
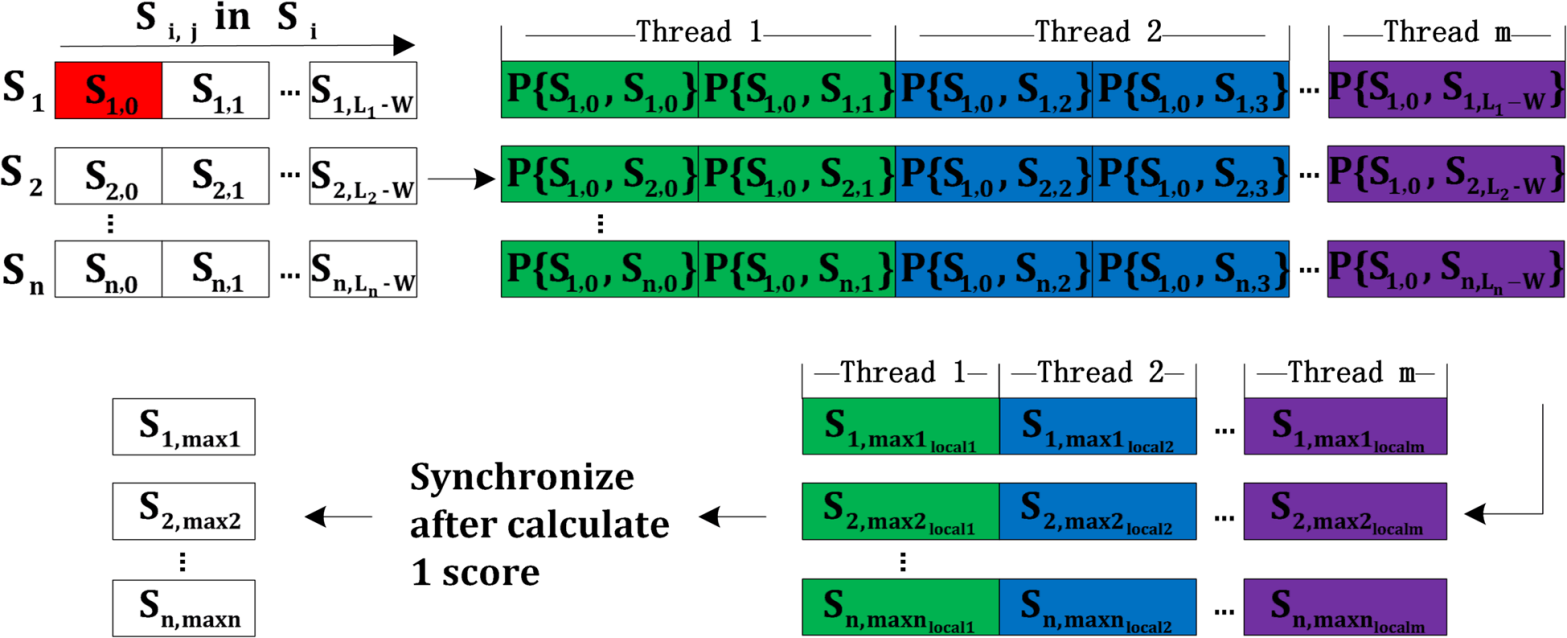
Process of probability score computation algorithm

As observed in Eq. (3), the score computation for the *j*th iteration depends on the scores for the (*j-1*)th iteration. Therefore we create two vectors to store the scores for two iterations using a simple cyclic vector swapping method. In *j*th iteration, vector *A* as input stores the score for (*j-1*)th iteration and vector *B* as output stores the score for *j*th iteration. In next iteration, the two vectors are swapped, vector *B* serves as input and vector *A* serves as output. All threads in a group have to synchronize and swap the input and output score vector in each iteration. The detailed algorithm for the probability score computation is illustrated in Algorithm 2. And Fig. 3 shows the process of probability score computation.

By dividing threads into thread groups, the number of the threads which have to communicate to each other in one iteration is decreased and the overhead of synchronization is reduced in this method.

**Fig. 4:**
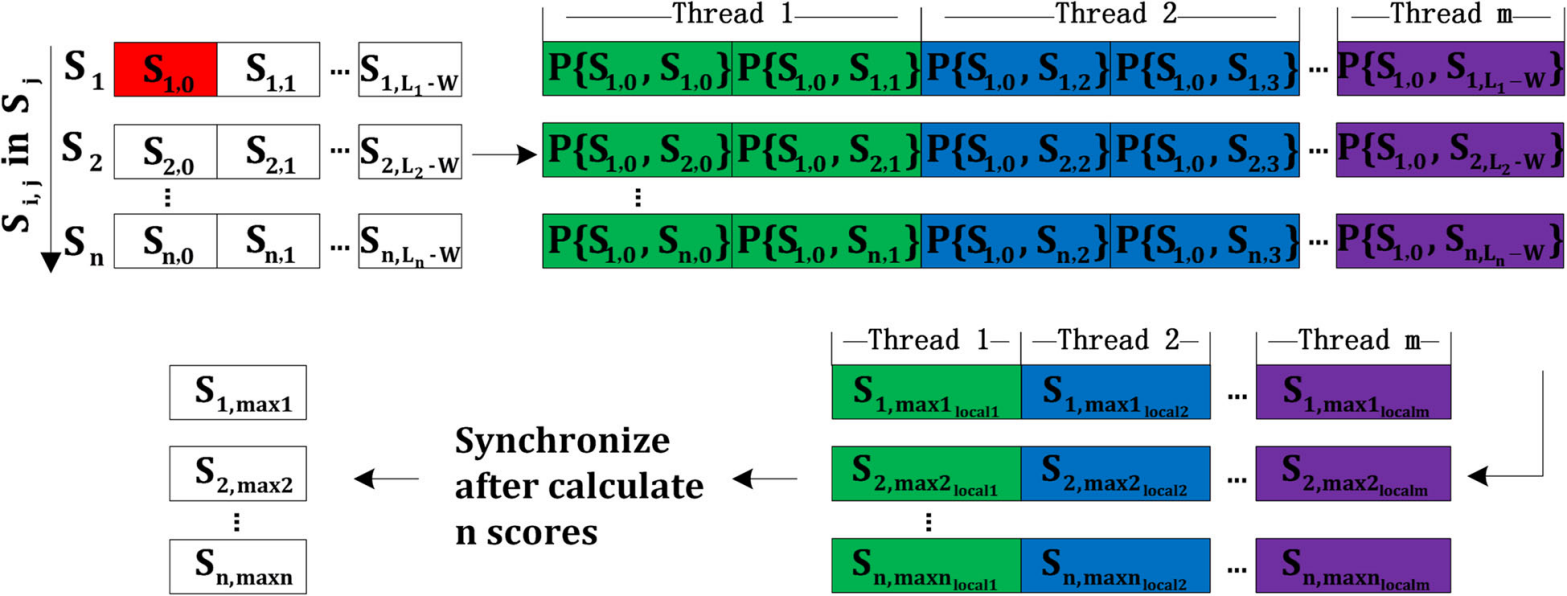
Process of probability score computation for datasets with a small number of long sequences algorithm

Using the method presented in Algorithm 2, it usually achieves high performance except for datasets with a small number of long sequences. To improve the performance of datasets with a small number of sequences, we present a new method which swaps the outermost loop and the second outermost loop presented in Algorithm 2. The computation of scores {*P*(*S*_*i*, *j*_, *S*_*k*, *l*_)} for sequence *S*_*i*_ (*1*≤*i*≤*n*) in *S* against any sequence *S*_*k*_ are independent of each other. After swapping the loops, for a given position *j*, scores {*P*(*S*_*i*, *j*_, *S*_*k*, *l*_)} for all substrings *S*_*i*, *j*_ (*1*≤*i*≤*n*) against any sequence *S*_*k*_ are computed in one iteration. Although the workload of each thread is increased, it does not influence too much since the number of sequences is small. On the contrary, the times of synchronization are decreased, which reduces overhead of synchronization and improves the performance. The detailed algorithm for the probability score computation for datasets with a small number of long sequences is illustrated in Algorithm 3. And Fig. 4 shows the process of the algorithm. In the outermost loop of Fig. 4, scores *P*(*S*_*i*, *j*_, *S*_*k*, *l*_) are computed in the order of j instead of in the order of i. And the threads synchronize after calculate n scores instead of one score.

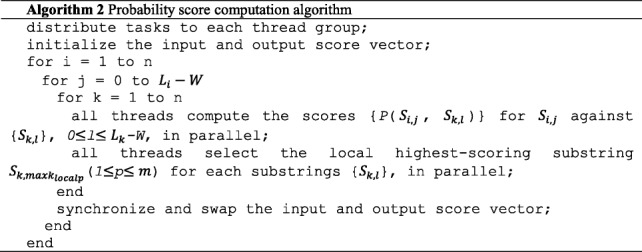

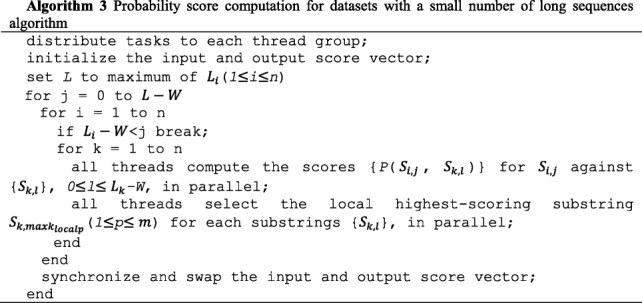





However, the algorithm presented in Algorithm 3 may not work well for datasets with a large number of sequences, since the large number of sequences contributes to large workload which is not appropriate for each thread. So to achieve the highest performance, the number of sequences is checked at first to decide to use which algorithm.

After probability score computation part, the local highest-scoring substring ${S}_{k,{maxk}_{localp}}$ for each set of substrings {*S*_*k*, *l*_} has been selected. In highest score sortation part, each thread is assigned to select the highest-scoring substring *S*_*k*, *maxk*_ for each *S*_*k*_ depending on the local highest-scoring substrings {${S}_{k,{maxk}_{localp}}$} and sort the *nsites0* highest-scoring substrings {*S*_*k*, *maxk*_} in decreasing order of scores to determine the potential starting points. At the end, the hash map and starting point heap are updated serially.

During starting point searching stage, the hybrid CPU/MIC computing framework is used only in the probability score computation part. The framework is not used in highest score sortation part since there are some complex structures like hash map in this part which are hard to be offloaded to MIC.

### Improved iteration updating strategy

**Fig. 5:**
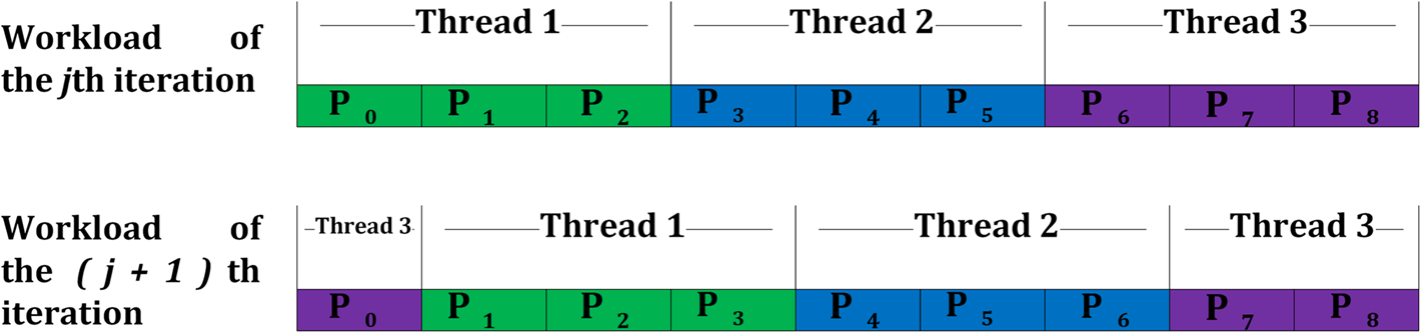
Improved task partitioning strategy

MEME Suite has defined an option “-revcomp” [12] to consider both the given strand and the reverse complement strand when searching for motifs in a complementable alphabet (*ie* DNA). Without this option, the algorithm will search for motifs of complementable alphabets on the given strand only. In this paper, we present an improved iteration updating strategy to avoid synchronization and improve the performance when option “-revcomp” is not selected. As observed in Eq. (3), the score computation for the (*j + 1*)th iteration depends on the scores for the *j*th iteration. Assume that thread *A* computes *q* scores {*P*(*S*_*i*, *j*_, *S*_*k*, *l*_)} for *S*_*i*, *j*_ against {*S*_*k*, *l*_},*1*≤*k*≤*n* and *0*≤*l*≤*q* − 1. In this method, scores {*P*(*S*_*i*, *j*_, *S*_*k*, *l*_)}, *1*≤*k*≤*n* and *0*≤*l*≤*q* − 1, are computed by thread *A* in the first iteration. In the next iteration, the task is changed to scores {*P*(*S*_*i*, *j*_, *S*_*k*, *l*_)}, *1*≤*k*≤*n* and *1*≤*l*≤*q*, so that the scores computed by thread *A* in next iteration only depend on the scores computed in this iteration by itself. In this way, each thread does not need to transfer scores to each other in one iteration, which avoids synchronization. The strategy is shown in Fig. 5.

To get good performance out of the Intel MIC Architecture, applications need to take advantage of the 512-bit vectorization unit. Verctorization [15] advances many data in the array by just one instruction, which is the most important method to obtain better performance.

In this method, the hotspot, computation of the probability scores {*P*(*S*_*i*, *j*_, *S*_*k*, *l*_)} presented in Eq. (3) is vectorized. By vectorizing the hotspot, we have achieved a speedup of 2 for the overall runtime.

## Results and discussion

### Data and platform

Our experimental platform is a high-performance server which consists of 8 Xeon E7–8800 v3 18 core CPU processors with 2 Xeon Phi 3120 57 core coprocessors and 2 K40 M GPU. Other specifications of the experimental platform are listed in Table 1. And Intel C++ Compiler (icc) 16.0.3 is used to compile the MIC-MEME.

**Table 1:** Platform specifications

	CPU	MIC
Clock Frequency(GHz)	2.5	1.1
VPU width(bits)	256	512
L1/L2/L3 Cache(KB)	32/256/2560	32/512/−
Memory Size(GB)	2048	6

To evaluate the scalability of our algorithm with respect to dataset size, the following datasets with different numbers of sequences and base pairs (bps) were used during this experiment: the mini-drosoph dataset and three datasets of human promoter regions consisting of 100, 200 and 400 sequences of lengths 5000 base-pairs each (called HS_100, HS_200 and HS_400, respectively). Table 2 shows the detailed specifications of datasets.

**Table 2:** Datasets specifications

Datasets	Number of sequences	Min(bps)	Max(bps)	Total(bps)
mini-drosoph	4	12,850	297,266	499,297
HS_100	100	5000	5000	500,000
HS_200	200	5000	5000	1,000,000
HS_400	400	5000	5000	2,000,000

### Speedup

To evaluate the performance of MIC-MEME, the following parameters were used: “-dna -mod zoops –revcomp”, “-dna -mod oops –revcomp”, “-dna -mod zoops” and “-dna -mod oops”. They include all the situations of ZOOPS and OOPS models.

**Fig. 6:**
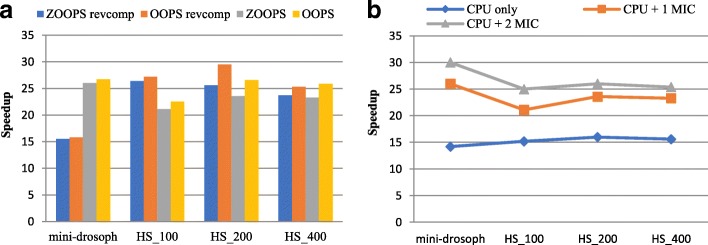
Performance and Scalability of MIC-MEME. (**a**) Performance of MIC-MEME with different datasets and parameters. (**b**) Scalability of MIC-MEME with respect to the number of MIC

We just evaluated the performance of MIC-MEME running on the server with 24 processes on CPU and 224 processes on a single MIC. The ratio of workload on CPU and one MIC is 1/3. Fig. 6a demonstrates the speedups of MIC-MEME using these parameters for all datasets.

As Fig. 6a shows, when considering both the given strand and the reverse complement strand, MIC-MEME achieves an average speedup of 22.8 for the overall runtime (with the highest of 26.4 and the lowest of 15.5) using ZOOPS model and achieves an average speedup of 24.5 for the overall runtime (with the highest of 29.5 and the lowest of 15.8) using OOPS model. When considering the given strand only, MIC-MEME achieves an average speedup of 23.5 for the overall runtime (with the highest of 26 and the lowest of 21.1) using ZOOPS model and achieves an average speedup of 25.4 for the overall runtime (with the highest of 26.7 and the lowest of 22.5) using OOPS model. Note that, MIC-MEME produces the same results as sequential MEME using these parameters and the results demonstrate increasing trends as the datasets become larger.

And in addition to the scalability with respect to dataset scale, we further evaluated the scalability of our algorithm with respect to the number of MIC. The test was performed on CPU only, CPU with one MIC and CPU with two MICs respectively. Note that, the ratio of workload on CPU with one MIC is 1/3 and the ratio of workload on CPU with two MIC is 1/1. The parameter “-dna -mod zoops -revcomp” was used. The test result is shown in Fig. 6b.

It can be seen that the average speedup of MIC-MEME running on CPU only is 15.3 (with the highest of 16 and the lowest of 14.2), the average speedup of MIC-MEME running on CPU with one MIC is 23.5 (with the highest of 26 and the lowest of 21.1) and the average speedup of MIC-MEME running on CPU with two MIC is 26.6 (with the highest of 30 and the lowest of 25). Therefore, the average speedup of our algorithm increases as the number of MIC increasing, which demonstrates the good scalability of MIC-MEME respect to the number of MIC.

**Fig. 7:**
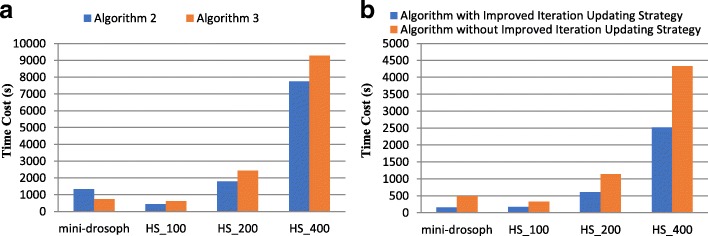
(**a**) Performance of Algorithm 2 and Algorithm 3. (**b**) Performance of Improved Iteration Updating Strategy

We also evaluated the performance of Algorithm 2 and Algorithm 3 with datasets that have different numbers of sequences. In this test, the parameter “-dna -mod zoops -revcomp” was used. Fig. 7a shows the performance of Algorithm 2 and Algorithm 3. As observed, Algorithm 2 runs faster than Algorithm 3 except for the mini-drosoph dataset. But Algorithm 3 is 1.8 times faster than Algorithm 2 when handling mini-drosoph. Note that, the number of sequences of mini-drosoph is only 4 and the numbers of sequences of other datasets are larger than 100. The result proves that Algorithm 3 performs better than Algorithm 2 when handling datasets with a small number of sequences. However, Algorithm 3 does not work well for datasets with a large number of sequences such as HS_100, HS_200 and HS_400. To achieve the highest performance, the number of sequences needs to be checked to decide which algorithm will be used.

To prove the merit of improved iteration updating strategy, the performance of the algorithm with improved iteration updating strategy was compared with the performance of the algorithm without improved iteration updating strategy. The parameter used in this test is “-dna -mod zoops”. As Fig. 7b shows, the algorithm with improved iteration updating strategy is average 2.2 times faster than the algorithm without improved iteration updating strategy. The result demonstrates the effectiveness and advantage of improved iteration updating strategy.

### Comparing with state-of-arts methods

**Fig. 8:**
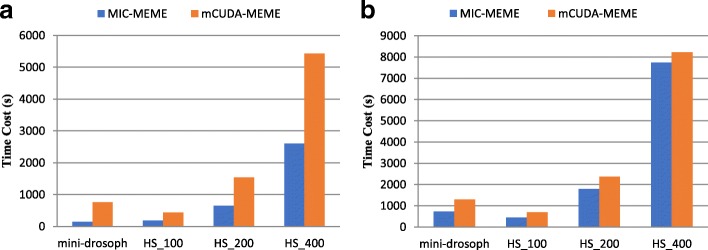
Performance of mCUDA-MEME and MIC-MEME. (**a**) performance of mCUDA-MEME and MIC-MEME without “revcomp” option. (**b**) performance of mCUDA-MEME and MIC-MEME with “revcomp” option

Furthermore, we have compared the performance of MIC-MEME with three other state-of-arts methods: EXTREME [16], mCUDA-MEME and BoBro2.0 [17]. EXTREME is a recent improvement of MEME which applies the online EM algorithm to discover motifs and uses the same model as MEME. BoBro [18] is an algorithm for prediction of cis-regulatory motifs in a given set of promoter sequences. And BoBro2.0 is an improved version of BoBro. To compare with the other state-of-arts methods, MIC-MEME was evaluated running on CPU with one MIC, mCUDA-MEME was benchmarked on our server with CPU and one K40 m GPU, BoBro2.0 and EXTREME were benchmarked on our server with CPU only. As observed, MEME algorithm searches for motifs which contain seven kinds of lengths during one execution. Therefore, we make BoBro2.0 search for motifs with seven kinds of lengths respectively to make the workload of BoBro2.0 similar to the MIC-MEME’s. Because mCUDA-MEME and MIC-MEME are both based on MEME algorithm, they have the similar workload when using the same parameter. In this test, parameters “-dna -mod zoops -revcomp” and “-dna -mod zoops” were used to evaluate mCUDA-MEME and MIC-MEME. Fig. 8 shows the performance of mCUDA-MEME comparing with MIC-MEME. And Table 3 shows the time cost of each method with different datasets.

**Table 3:** Time cost of each method with different dataset. The time is given in seconds(s)

		mini-drosoph	HS_100	HS_200	HS_400
MIC-MEME	without “revcomp”	140 s	185 s	642 s	2608
	with “revcomp”	731 s	444 s	1788s	7744 s
CUDA-MEME	without “revcomp”	760 s	437 s	1535s	5428 s
	with “revcomp”	1298s	688 s	2362 s	8225 s
BoBro2.0		segment fault error	143,246 s	463,802 s	memory corruption error
EXTREME		1453s	4440 s	11,189 s	21,592 s

As Table 3 shows, the time spent by BoBro2.0 and EXTREME is much longer than the time spent by mCUDA-MEME and MIC-MEME, especially BoBro2.0. Besides, during the execution of BoBro2.0, segment fault error occurred when the searching with mini-drosoph and memory corruption error occurred when the searching with HS_400. However, BoBro2.0 and EXTREME are both written in scripting languages, which contributes to low efficiency. And we also find that EXTREME spends much more time on generating seeds which is the hotspot of the program.

As observed, using ZOOPS model without “revcomp” option, MIC-MEME is average 3 times faster than mCUDA-MEME (with the highest of 5.4 and the lowest of 2.1) and MIC-MEME is average 1.4 times faster than mCUDA-MEME (with the highest of 1.7 and the lowest of 1.1) using ZOOPS model with “revcomp” option. In conclusion, MIC-MEME outperforms mCUDA-MEME when considering the given strand only because of the improved iteration updating strategy which avoids synchronization. When considering both the given strand and the reverse complement strand, MIC-MEME just runs a little faster than mCUDA-MEME due to synchronization overhead especially for HS_400 dataset. Furthermore, MIC-MEME absolutely outperforms BoBro2.0 and EXTREME. However, MIC-MEME might not be able to work well in the situation where the number of sequences of datasets is extremely large because of synchronization and the limitation of computing resources of a single node. Maybe MIC together with MPI, Spark or Hadoop could solve this problem [19], which would be our future works.

## Conclusions

Discovering motifs in biological sequences is a crucial problem. For example, in DNA sequences, the phosphodiester oligonucleotide containing a newly identified CpG DNA motif can strongly stimulate CD86, CD40, CD54, and MHC class II expression, IL-6 synthesis, and proliferation of primary human B cells [20]. And a motif in DNA sequences may result in DNA binding sites [21, 22]. In protein sequences, C-terminal microbody targeting motifs are known to be targeted to microbodies [23] and LXXLL motif present in RIP-140, SRC-1 and CBP is necessary and sufficient to mediate the binding of these proteins to liganded nuclear receptors [24]. And in RNA sequences, motif may be able to bind ATP [25] or be recognized by RNA-binding proteins [26].

In this paper, we have improved MEME algorithm and present MIC-MEME to make use of the powerful compute capability of MIC. MIC-MEME primarily focuses on parallelizing the starting point searching stage and an improved iteration updating strategy is presented. It has achieved average speedups of 26.6 for ZOOPS model and 30.2 for OOPS model for the overall runtime on the server with 24 processes on CPU and 224 processes on multiple MICs. Furthermore, our algorithm shows good scalability with respect to dataset size and the number of MICs. And MIC-MEME has been compared favorably with mCUDA-MEME on the server with K40 m GPUs to prove its merit. With the increase of biological data, we hope the efficient motif discovery of MIC-MEME will be able to help the bioresearch work. However, the synchronization and limited computing resources of a single server node still constrain the efficiency of MIC-MEME. In the future, we will focus on parallelizing MIC-MEME on multiple nodes cluster.
